# Strong community-based health systems and national governance predict improvement in coverage of oral rehydration solution (ORS): a multilevel longitudinal model

**DOI:** 10.7189/jogh.10.010503

**Published:** 2020-06

**Authors:** Althea Andrus, Robert Cohen, Liliana Carvajal-Aguirre, Shams El Arifeen, William Weiss

**Affiliations:** 1 Alutiiq, US Department of State contractor, Washington, DC, USA; 2 CAMRIS International, Inc., USAID contractor, Bethesda, Maryland, USA; 3 UNICEF, New York, New York, USA; 4 icddr,b, Dhaka, Bangladesh; 5 Department of International Health, John Hopkins University, Baltimore, Maryland, USA; 6 Sustaining Technical and Analytic Resources (STAR) Project, Public Health Institute, USAID Contractor, Washington, DC, USA

## Abstract

Diarrheal disease remains a leading cause of child death globally, especially in low and middle-income countries. Use of oral rehydration solution (ORS) for treatment of diarrhea in children, a very cost-effective intervention, remains below 50% in many countries. Here we use a multi-level longitudinal model to reveal important predictors of ORS use at the national level. The findings suggest that increasing government effectiveness along with increased implementation and affordability of community-based health programs can lead to substantial increases in ORS use. Key informant interviews with national health leaders in countries that significantly improved ORS coverage support these quantitative findings.

Diarrhea remains a leading cause of death among children under five [1,2], disproportionately affecting children in low- and middle-income countries [3]. One of the most cost-effective interventions for preventing death from a case of diarrhea is to treat it with oral rehydration solution (ORS), which reduces diarrhea-specific mortality by up to 93% [4]. While there are over 50 years of evidence demonstrating the effectiveness of ORS, underutilization of ORS continues in many countries with a high burden of child mortality from diarrhea [5]. The proportion of children with an episode of diarrhea who receive ORS has increased unevenly since first introduced in the 1980s [3,6] and remains below 50% in many low- and middle-income countries (LMICs) despite new formulations or low-osmolarity ORS that make it more acceptable to children, even if not dehydrated. Scaling up the use of ORS to treat diarrhea is one of the most cost-effective ways a country can accelerate the reduction of preventable child deaths. If not done, it represents a potential missed opportunity for helping a country achieve the child mortality target of Sustainable Development Goal 3: an under-5 mortality rate of 25 per 1000 live births or lower [7]. In order to help countries scale up the use of ORS for treatment of childhood diarrhea, we seek to identify factors that are predictive of ORS use on a population level, especially those factors that are sensitive to change from investments and efforts of donors, governments and communities.

Prior studies of caretakers’ management of diarrheal episodes and their care-seeking behaviors have improved understanding of what predicts ORS use in several countries. These predictors include the following: (1) community empowerment and engagement [2,3,8-13], particularly among vulnerable populations [6]; (2) the mother’s level of education [14-16]; (3) access to health care [13-15]; and, (4) education among medical professionals and/or caregivers [9,10,13,17-25]. Although the studies identify predictors of ORS use across several countries, generalizable knowledge about the predictors of ORS use globally is limited, and systematic literature reviews have highlighted this gap in knowledge [3,13,14]. Bridging this gap is essential to identify the policies and population level programs that will lead to increased ORS use and fewer preventable child deaths.

This study explores the national-level predictors of ORS use globally through a quantitative model similar to the one used in the *Success factors study for reducing maternal and child mortality* [24]. That study sought to explain why some countries have reduced maternal and under-five mortality more quickly than comparable ones. The quantitative part of the *Success factors study* analyzed over 250 different health determinants and found that multi-sectoral improvements in over a dozen policy areas – including immunizations, water and sanitation, and women’s education – contributed additively and synergistically to under-five mortality reduction [26-28].

We hypothesize that we can identify a set of quantitative national level predictors, measuring factors from within and outside the health system, that would enable countries to accelerate increases in ORS use at the population level (ORS coverage) if addressed alone or in combination. The value-add of this study of ORS coverage is 3-fold. First, this study includes more countries, potential predictor variables, and years of observation compared to earlier studies, and therefore the findings should be more generalizable. A second value-add is the use of a longitudinal multilevel model to compare changes in potential predictor variables with changes in ORS coverage in the same time periods. Third, this method generates estimates of the potential impact on ORS coverage from changes in identified predictor variables, aiding policymakers in setting targets.

## METHODS

The analysis presented here is a secondary data analysis of publicly available data, primarily from nationally representative household surveys.

### Period

We used data from 1996-2016 for two main reasons. First, the beginning of this period approximately matches the launch of the Integrated Management of Childhood Illness (IMCI) initiative in 1996 by the World Health Organization [29], with an emphasis on case management of diarrhea. Second, the period also overlaps considerably with the efforts of many countries to achieve the Millennium Development Goals. Third, some of the independent variables, most notably health financing and governance, were first reported in 1995 and 1996, respectively. The end, 2016, matches the year of the most recently available household health survey used in the analysis (such as the Demographic and Health Survey or DHS, and the Multiple-Indicator Cluster Survey or MICS [30,31]). Fourth, no studies have examined factors associated with ORS coverage on a large scale with many potential factors in this period.

### Dependent variable

The dependent variable is the percent of children with diarrhea in the last two weeks who were given ORS as reported by caretakers primarily published in the DHS or MICS between 1996 and 2016 [30,31].

### Independent variables

To identify the independent variables, we constructed a data set of potential predictors from the following sources: (1) predictors identified in prior studies of ORS coverage; (2) variables that were significantly associated with under-5 mortality reduction in the *Success Factors study* [26-28]; and (3) variables reflecting the timing of national child health policies. Using these criteria, we tested 34 indicators as independent variables; all downloaded from the USAID Idea database [32] (**Table 1**).

**Table 1:** Results of univariable analysis*

Development Sector	Indicators
**Wealth**	GDP per capita in constant 2010 US$ (natural log)§
**Demographics**	Total fertility rate †
	Percent of population in urban areas
**Governance**	World Bank political stability index§
	World Bank government effectiveness index‡
	Percent of parliamentary seats held by women‡
	Percent of women with access to TV, radio, or newspaper
**Water, Sanitation, Hygiene**	Percent of population with improved water access†
	Percent of population with improved sanitation access
**Nutrition**	Percent of population with height-for-age z scores<-2 SD (stunting)
	Percent of population with a body mass index <18.5 (underweight)
	Percent of population with weight-for-height z scores<-2 SD (wasting)‡
	Percent of population with weight-for-height z scores<-3 SD (severe wasting)§
**Education**	Average years of education, among females age 20-24 y§
	Percent of net secondary school enrollment, among females
	Percent of net secondary school enrollment, among females lagged five-years
	Percent of net primary school enrollment, among females§
	Percent of net primary school enrollment, among females lagged ten-years§
**Health System**	Policy recommending management of pneumonia in the community or at home by a trained provider (yes, no)
	Percent of pregnancies with at least one antenatal care visit with a qualified medical practitioner§
	Percent of pregnancies with at least four antenatal care visits‡
	Percent of children under-five that slept under an insecticide treated net last night
	Percent of births that were in a qualified medical institution
	Percent of children under-five that had upper-respiratory symptoms and sought care from a qualified medical practitioner (care-seeking for pneumonia)†
	Percent of children under-five that have received their DPT3 immunization†
	Percent of births that were attended by a skilled birth attendant
	Percent of children under-five that had a fever and sought care from a qualified medical practitioner (care-seeking for fever)
	Number of physicians per 1000 population
	Percent of births via caesarean section‡
	Percent of newborns that were checked by a qualified medical practitioner within 48 h of birth
**Health Financing**	Out-of-pocket health expenditure as a percent of total health expenditure§
	Health expenditure per capita in constant 2011 international $ (natural log)§
	External health spending as a percent of government health expenditure‡
	Government health expenditure as a percent of GDP§
	Total health expenditure as a percent of GDP

GDP – gross domestic product, SD – standard deviation, DTP3 – diphtheria-tetanus-pertussis

*With the exception of ‘Policy recommending management of pneumonia in the community or at home by a trained provider’, all data was downloaded from the USAID Idea Database [32]. Health financing, wealth, and three of the governance indicators (stability index, governance index, and parliamentary seats) were originally sourced from the World Bank. The health system, water and sanitation, nutrition, and the remaining governance indicator (women’s access to radio, TV, and news) were originally from Demographic and Health Surveys. The Education variables were originally sourced from UNESCO. The policy variable was originally sourced from WHO.

†*P* < 0.1.

‡*P* < 0.05.

§*P* < 0.01.

### Policy variables

The WHO Global Maternal Newborn Child and Adolescent Health Policy Indicator Survey includes information about adoption of WHO recommendations for national health policies and guidelines related to maternal, newborn, child, and adolescent health [33]. We screened the survey database for child health policies that were either directly relevant to ORS or relevant to predictors of ORS identified in the literature. A requirement for longitudinal analysis techniques was information about the years in which a policy was in place or not. The one policy that met this requirement was approval for childhood pneumonia to be managed with antibiotics in the community or at home by a trained provider [33]. This policy is known as community case management of pneumonia and may be a component of an integrated community case management approach (iCCM). We included this policy as a potential independent variable as a proxy for increased national interest in preventing child deaths through community programs. Because pneumonia and diarrhea are leading causes of death for children under the age of five in low and middle income countries, we believe that improvements in how pneumonia in children is being managed at the community level reflects the national interest and political will to address child mortality overall at the community level, including efforts to prevent deaths from diarrhea. For analysis purposes, the information about this policy variable was converted to a Present/Not Present dummy variable for each year from 1996- 2017 in our analytical data set.

### Countries included in the analysis

All 193 United Nations member states were considered eligible for the analysis. We weighted each country equally; such that large countries did not dominate the analysis. Alternatively, to prevent the analysis from being distorted by small countries, we excluded countries where the under-5 population averaged under 150 000 children each year during the study period (with a range of 150 000 to 126 000 000 [34]). The cut-off naturally emerged in the population distribution and excludes the smallest countries that can be problematic in model estimation. We included the 103 remaining countries that had at least one population level household survey that measured ORS coverage since 1996. Of these, per the World Bank’s definition in 2017, 30 were low-income countries, 43 were lower-middle income countries, and 30 were upper-middle income countries.

### Data adjustments

We adjusted the data and, when necessary, transformed it to facilitate modeling. The following variables were transformed to log values to improve model fit: GDP per capita (constant 2010 USD), total health expenditure per capita (constant 2011 international $) [32]. In addition to using primary and secondary school net enrollment rates, we also used enrollment rates lagged by ten years, for primary enrollment, and five years, for secondary enrollment, to test for inclusion in the final model [32]. ORS coverage is normally distributed, and therefore, we did not need to transform the dependent variable [30,31].

Independent variables were missing data in country/y when Demographic and Health Surveys had not been conducted. In addition, because education enrollment rates were lagged, there were no data for the most recent country/y. With the exception of the policy variable for Community Case Management (CCM) for pneumonia, covariate data were not missing at random; thus, we elected not to use imputation to fill in missing data for the other variables. For countries that had data available for some years, but not all, we linearly interpolated these other variables, and when data were not available for recent years, we created a forecast using a five-year moving average of the rate of change in the indicator.

For the missing CCM data, because linear interpolation was not possible, we imputed data for four countries – Dominican Republic, Jordan, Nicaragua, and Philippines–which had no reported data. The data were imputed using linear regression with the policy variable as a dependent variable and with independent variables that had at least a 0.25 correlation with available data. These independent variables were time, percent of parliamentary seats held by women, and DPT immunization among children 12-23 months. Although imputation is a valid method to fill in missing dummy data [35] when data are missing at random, these data were not missing at random. Therefore, we interpret models using imputed data with caution and only if the results concur with models without imputed data.

### Longitudinal multi-level model

#### Model structure

We considered the baseline model with ORS as the dependent variable and year as the independent variable. A Hausman test [36] revealed that a random-effects model was preferable because it was consistent and more efficient than a fixed-effects model (Table S1 in the **Online Supplementary Document**). Robust standard errors were used to account for mild violations of underlying assumptions. We placed no constraints on the covariance between random effects. The model had random intercepts for country and World Bank region, unstructured covariance, and no autocorrelation across time. The model also included a random coefficient for time (year of survey). The equation is:

y_ij_ = β_0_ + β_1_ × t_j_ + βX_ij_ + α_k_ + μ_i_ + γ_i_ × t_j_ + ϵ_ij_

Where *y_ij_* is the percent of children under five that had diarrhea in the last two weeks who were treated with ORS in country *i* in year *j, X_i_*_j_ is a matrix of independent variables in country *i* in year *j*, *α_k_* is a random intercept for region *k*, *μ_i_* is a random intercept for country *i,* and *γ_i_* is a random coefficient for country *i*.

All analyses were conducted in STATA (StataCorp., College Station TX, USA) 14 using the *xtmixed* command [37]. The relevant Stata code was:

*xtmixed ors time [variables] if year >1995 || region: || country: time, cov(uns) vce(robust) mle*.

### Selection of covariates and parameters to include in the final model

We initially tested all 36 variables separately in the above model. We considered variables that were statistically significant at the *P* < 0.10 level in the univariable analysis as candidates for the multivariable model.

To construct the multivariable model, stepwise regression was conducted using the covariates that were found to be significant in the univariable analysis (excluding the education covariates initially, which were reported less than the other variables). After imputation and interpolation, there were 235 country-years data points for variables which were significant in the univariable analysis. We therefore restricted the multivariable analysis to these 235 country-years for model comparisons. We selected a final model based on Akaike Information Criterion, or AIC [37] because it does not penalize model complexity as much as the Bayesian Information Criterion. Once we had a final model, we ran the regression for both the restricted data set of 235 data points and all 300 available data points for those variables. For this final model of 300 data points, we then tested whether the random coefficient added predictive value (Table S2 in the **Online Supplementary Document**). We continued iteratively adding variables to the model until we could no longer find a model that had an improved AIC.

### Quantifying the impact of independent variables

With our final model of the full data set, we used the coefficients and their standard errors to predict the absolute annual increase in ORS coverage based on the values of annual improvement of the independent variables at the median, 75^th^ percentile, and 90th percentile of the sampled country-year pairs. We constructed 95% confidence intervals by multiplying 1.96 by the combined standard error for this estimate; which is as follows:


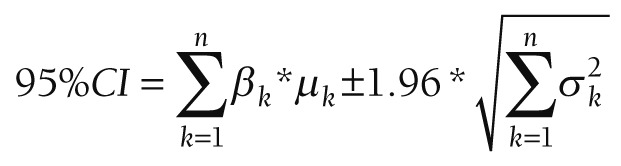


Where k is the *n*^th^ parameter, *μ* represents the 50^th^, 75^th^, or 90^th^ percentile of the *n*^th^ parameter, and *σ^2^_k_* is the standard error of *β_k_* in the final multivariable model.

### Goodness of fit

Calculating the overall goodness of fit for multilevel models can be a challenge. We utilized the traditional method of calculating R^2^ using the equation *Σ(^y–y_i_)^2^/Σ(yi–ȳ)^2^*, because two recent examinations of methods for calculating goodness of fit in complex models has been shown that this method has less than a 1% bias of the actual value [38,39].

Multiple analyses were conducted to assess if the model’s results were impacted by missing data. After a final model was developed, we regressed the same model with a restricted data set with no missing values for education and the CCM policy. This restricted data set had 157 observations. The model was also tested with an unrestricted data set of 338 observations.

After these analyses were conducted, we applied bootstrapping methods with 100 simulations for the model with both the 235 observation data set and the full unrestricted model of 338 observations.

### Key informant interviews

To better interpret the results of our analysis, we conducted open-ended data gathering with key informants. Key informants were purposively sampled. We interviewed persons identified as being knowledgeable about child health programs in countries that experienced changes in ORS coverage greater than 10 percentage points (positive or negative) between two household surveys. The countries of interest were the following: (1) Burundi – an increase in ORS coverage between 2000 and 2005 of 24 percentage points from 11% to 35%; (2) Ghana – an increase of 17 percentage points from 2006-2008 (from 29 to 46 percent) followed by a decrease of 11 points in 2008-11 period from 46 to 35 percent, and then an increase of 16 points from 2011 to 2014 (35 to 51 percent); (3) Guinea-Bissau – a decrease in ORS coverage from 2000-06 of 16 points (from 39 to 23 percent) with a later increase of 48 percentage points from 2010-14 (from 19 to 67 percent); (4) Niger – an increase of 26 percentage points from 19% in 2006 to 45% in 2012; (5) Swaziland – an increase of 19 percentage points from 2000-07 (from 66 to 85 percent), a decrease of 28 percentage points from 2007-10 (from 85 to 57 percent), followed by an increase of 27 percentage points from 2010-14 (from 57 to 84 percent); and, (6) Bangladesh – an average annual increase of five percentage points throughout the period. We identified key informants in the specific countries through contact with UNICEF country offices or personal relationships with the authors. UNICEF staff in countries and bilingual USAID colleagues translated the instrument to French or Portuguese. Main questions were followed by probing questions provided in the instrument that was emailed to informants and returned by email with their answers. These experts were asked to respond to an open-ended question about what they believed were the main reasons why ORS coverage changed in their country in the periods referenced above.

## RESULTS

### Changes in ORS coverage

**Figure 1:**
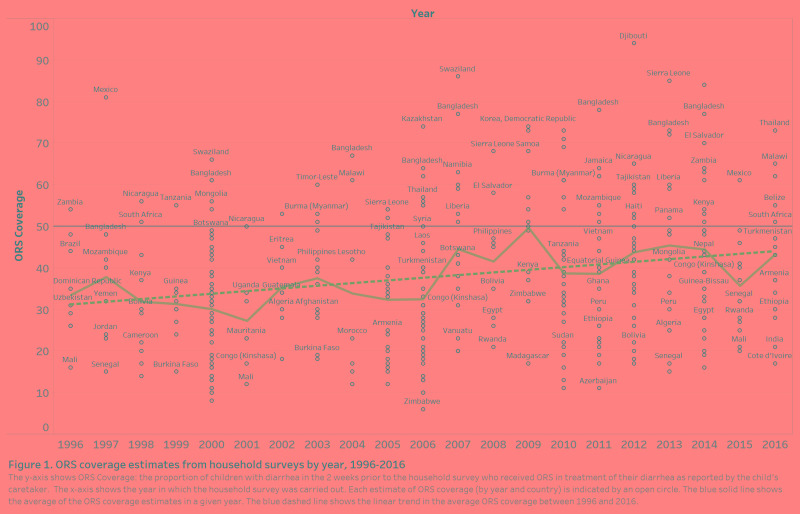
Oral rehydration salt (ORS) coverage from household surveys by year, 1996-2016. The axis shows ORS coverage: the proportions of children that had diarrhea in the two-weeks prior to the household survey who received ORS in treatment of their diarrhea as reported by the child’s caretaker. The x-axis shows the year in which the household survey was carried out. Each estimate of ORS coverage is (by year and country) is indicated by an open circle. The blue solid line the average ORS coverage estimates for the given year. The blue dashed line shows the linear trend in the average ORS coverage between 1996 and 2016.

Increases in ORS coverage between survey years are uneven. The absolute annual change in ORS coverage between surveys is normally distributed with a mean of 0.66 percentage points per year (**Figure 1**, n = 307, standard deviation = 2.9, range -12.5 to 14.5).

### Univariable analysis

Improved ORS coverage was significantly associated with 20 of the 36 independent variables at the *P* < 0.10 level (**Table 1**; Table S3 in the **Online Supplementary Document**). These included five of twelve health system variables and four out of five health financing variables. Among the education variables, we found that primary school had a stronger correlation with ORS coverage than did secondary school. Three of the four governance variables showed a relationship with ORS coverage as did two of the four nutrition variables. Total fertility rates, GDP per capita, and access to improved water were also found to be significant in the univariable analysis.

### Multivariable analysis

In addition to time, the final model included four independent variables that were selected by AIC. Backward elimination was used to try to remove excess model parameters which did not add predictive value, but all model parameters remained in the model. The independent predictor variables in the final model are the following: time, government effectiveness index, care-seeking for symptoms of pneumonia, out-of-pocket expenditures, and four or more antenatal care visits (**Table 2**; Tables S4-5 in the **Online Supplementary Document**). The OLS R^2^ value was 0.90.

**Table 2:** Regression output of final multivariable models*

	Final model 1	Final model 1	Final model 2	Final model 2	Final model 3	Final model 3	Final model 4	Final model 4	
Variables	**Restricted model**	**Bootstrap 100 reps**	**Unrestricted model**	**Bootstrap 100 reps**	**Restricted model**	**Bootstrap 100 reps**	**Unrestricted model**	**Bootstrap 100 reps**	
Care-seeking for pneumonia	0.152‡	0.152	0.173‡	0.173‡	0.210‡	0.210†	0.174§	0.174‡	
	(0.0379)	(0.103)	(0.0174)	(0.0199)	(0.0230)	(0.00448)	(0.0895)	(0.0111)	
Government effectiveness	5.804†	5.804†	4.413†	4.413§	4.323†	4.323§	5.589†	5.589†	
	(2.89 × 10^−7^)	(0.00634)	(0.000148)	(0.0656)	(2.13 × 10^−5^)	(0.0506)	(0.00584)	(0.00233)	
Out-of-pocket expenditures	-0.0945§	-0.0945	-0.0652‡	-0.0652	-0.0802‡	-0.0802	-0.137†	-0.137‡	
	(0.0778)	(0.187)	(0.0297)	(0.278)	(0.0290)	(0.200)	(0.00510)	(0.0169)	
ANC4+	0.0983§	0.0983	0.0817	0.0817					
	(0.0578)	(0.105)	(0.170)	(0.122)					
Year	0.387†	0.387†	0.388†	0.388†	0.436†	0.436†	0.525†	0.525†	
	(0.00134)	(0.00265)	(0.00372)	(0.00372)	(0.00018)	(00365)	(0.00132)	(4.89 × 10^−5^)	
Constant	21.05†	21.05†	18.45†	18.45†	20.84†	20.84†	23.18†	23.18†	
	(0.00525)	(0.000231)	(0.000142)	(0.000136)	(1.43 × 10^−9^)	(1.20 × 10^−5^)	(2.19 × 10^−6^)	(5.39 × 10^−6^)	
Observations	235	235	300	300	300	300	338	338	
Number of regions	5	5	6	6	6	6	6	6	
LogL	-859.7	-859.7	-1112	-1112	-1113	-1113	-1261	-1261	

*Robust *P-*value in parentheses.

†*P* < 0.01.

‡*P* < 0.05.

*P < 0.1

These results were robust to multiple sensitivity analyses. AIC preferred the same model whether we used the data set of 235 observations (Table S4 in the **Online Supplementary Document**), the more restricted data set of 157 observations with no missing values for education and CCM policy (Table S5 in the **Online Supplementary Document**), or an unrestricted data set of 300 or 338 observations in which we included all available country-years (**Table 2**). The education variables did not reach statistical significance when controlling for the other health system variables in the restricted data set. The CCM policy variable was excluded from the final model because it was not significant in the univariable analysis.

Statistical significance remained for all variables (except ANC4) after we applied bootstrapping methods with 100 simulations for the model with 235 observations and a full unrestricted model of 338 observations (**Table 2**).

### The variance of the random coefficient

**Figure 2:**
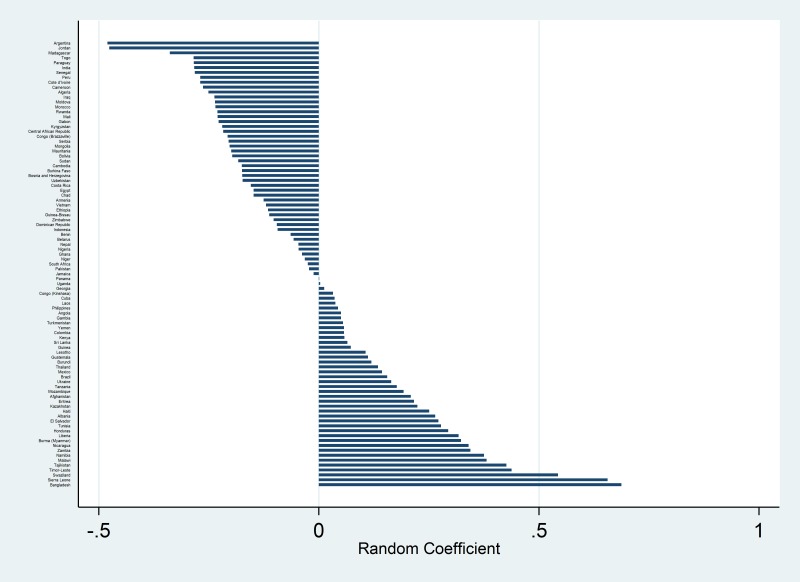
Random coefficients for time by country. The random coefficients for time in the figure represent the relevant speed at which countries improved oral rehydration salt (ORS) coverage (or not) after controlling for other factors. The countries with the most rapid increases are on the right side of the figure. The countries with the five highest values (reading from left to right) are Bangladesh, Sierra Leone, Swaziland, Timor-Leste and Tajikistan. The change predicted in ORS coverage from an increase in one year, after controlling for improvements in the other independent variables.

The random coefficient for time (*γ_i_*) represents the different speed at which countries improved ORS over time, after controlling for improvements in the other independent variables. For the 85 countries in the final model, *γ_i_* was normally distributed with a mean of 0, a standard deviation of .24, and a range of -.48 to.69 The countries with the five highest values for *γ_i_* were Bangladesh, Sierra Leone, Swaziland, Timor-Leste, and Tajikistan for whom *γ_i_* > 0.4 (**Figure 2**). These random coefficients were consistent with faster than average progress in these countries during the duration of the study (Figure 2).

### Potential improvements in ORS coverage given multi-sectoral progress

**Figure 3:**
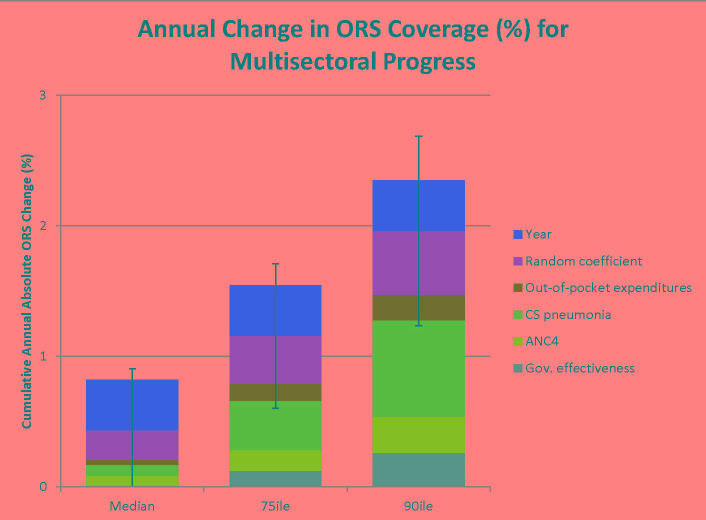
Annual predicted change in oral rehydration salt (ORS) coverage by level of multi-sectoral progress. Three potential scenarios for increasing ORS coverage are presented in the figure, depending on how much change there was in the independent variables in the final model. For example, if the independent variables increased from the median to the 75th percentile of the distribution, we would predict an average annual increase in ORS coverage of 1.5%, or an average annual increase of 2.3% if increased to the 90th percentile of the distribution. This represents a substantial change of 7.7% or 11.5% or a five-year period, respectively. The figure also shows the components of the predicted increases in ORS coverage based on the independent variables in the final model. The x-axis shows increases in covariate values at median, 75th percentile, and 90th percentile levels. The y-axis shows the predicted increase in ORS coverage following increases in covariates at the three levels. Covariates include time (year), out of pocket expenditures, care-seeking for pneumonia, four or more antenatal care visits, government effectiveness, and a random coefficient.

We used the coefficients from the final model to predict the increase in ORS that would happen in a country from changes in the independent variables in the model (Figure 3). For example, if all final model independent variables increased from the median to the 75^th^ percentile of the distribution, we predict on average an added annual improvement in ORS coverage of 1.5% (with a range of 1%-2.1%), or a mean of 7.7% over the typical 5-year inter-survey interval. If the independent variables increased to the 90th percentile, we would predict an added annual improvement of 2.3% (with a range of 1.6%-3%), or 11.5% over a 5-year period. These predicted improvements compare favorably to the current average annual rate of increase of 0.8%, or 4.1% over a 5-year period (see **Figure 3**). In addition, improvements in maternal and child health indicators at the primary care level (four or more antenatal care visits and care-seeking for pneumonia) predicted the largest increases in ORS coverage.

### Key informant interviews

After contacting eight informants in six countries, we only obtained responses from two informants each in Bangladesh, Burundi, and Guinea Bissau (total of six informants). Responses from these informants supported the use of the independent variables we had included in the final model and the results. Commonly mentioned reasons across informants suggest that the following factors were salient in affecting levels of ORS use: policies and programs supporting integrated management of childhood illness (IMCI) in health facilities, health promotion in the community, and treatment of childhood illness by community health workers (community case management).

“I think that from 2000 to 2010 [period of a drop in ORS coverage] the ORS were only available for the population through health centers, which may justify the low coverage. The increase in [ORS] coverage between 2010 and 2014 could be related to the initiation of CHWs activities in some regions of the country. As a result, there was an increase in the availability of ORS as well as in the knowledge of the population regarding the need for ORS in cases of diarrhea.” *(Informant from Guinea-Bissau)*

*[Period of ORS increase from 2010-2014]* “In 2013 the so-called PIMI project started, which is mainly focused on maternal and child health interventions at community level by Community Health Workers (CHWs). These are responsible for the treatment of simple diarrhea, using Oral Rehydration Salts (ORS) for that purpose.” *(Informant from Guinea-Bissau)*

“Training of health personnel at health center level in IMCI and community health workers at the community level in integrated CCM [were the main reasons ORS use increased between 2000 and 2005]. *(Informant from Burundi)*

*[Period of ORS increase from 2000-2005]* “The health promotion activities experienced a revival of interest with [leadership in health promotion that] was very active. These activities are effective when there is ownership at all levels. The central level provided impetus for implementation of these activities at the decentralized and operational level which in turn carried out sensitization at the community level. Thus, healthcare providers and communities were well aware of the benefits of ORS in treatment of childhood diarrhea and adopted the practice at scale. The fact that health personnel and caretakers of children understood the benefits and the impact of use of ORS in treatment of childhood diarrhea as well as the interest in the use of ORS for treatment childhood diarrhea was eliciting in the public health domain catalyzed the increase in coverage of ORS uptake.” *(Informant from Burundi)*

“The National Control of Diarrheal Diseases (CDD) program launched a nationwide ORT communication campaign in 1996 using a wide variety of communication channels to improve knowledge and awareness in the community on key rules of homecare- increased fluid, continued feeding and appropriate care-seeking during diarrhea… These campaigns reached a wide variety of community members and stakeholders including health providers and created an environment conducive to behavior change to adopt ORT and ORS as the tool for diarrhea management. The effect of these communication and promotional activities continued during the following years and created an increasingly sustained behavior of the community in using ORT and ORS during a diarrhea episode.” *(Informant from Bangladesh)*.

“The CDD program did a nationwide campaign in 1996 and continued following years to increase knowledge and awareness among the community people regarding oral rehydration therapy (ORT), continued feeding, proper care seeking for diarrhea. This multi- dimensional approach for increasing awareness among the community people, health care providers and other stakeholders was very successful and brought positive changes in attitude and practice of the population regarding treatment and care seeking behavior of diarrheal disease…. After closing of CDD program in 1998 Govt. of Bangladesh adopted WHO and UNICEF recommended Integrated Management of Childhood Illnesses (IMCI) as integrated strategy to fight child illness. Management of diarrhea was incorporated in that strategy. This helped significantly in institutionalization of ORS as a tool for management of diarrhea.” *(Informant from Bangladesh)*

One variable, stocks of ORS, was mentioned by several informants as a critical reason for increases and decreases in ORS use. However, this variable was not included in the final model because we could not identify publicly available data on ORS stocks for each year of the analysis and across most of the countries.

*[Period of ORS increase from 2000-2005]* “Health workers monitored the ORS stock levels closely and regularly made requisitions when the minimum stock level was attained on time to avoid stock-outs. Thus, ORS was available in health facilities on a regular basis and used to treat childhood diarrhea without any issues with stock-outs.” (Informant from Burundi)

*[Period of ORS increase from 2010-2014]* “…Therefore, the availability of ORS is guaranteed by UNICEF and distributed in five out of the eleven sanitary regions in the country. Same distribution is carried out at health centers level.” (Informant from Guinea Bissau)

“Later the govt. …did an agreement with SMC (Social Marketing Company), the largest social marketing company of Bangladesh. SMC started to produce ORS and marketed it throughout the country branded as ORSaline. ORS became available at the health facilities, local markets as well as to the depot holders. Depot holders were female community members, who were trained, used to collect ORS packets from the SMC at a subsidized price and distributed those to the community at market price. Hence ORS became available and accessible throughout the country.” *(Informant from Bangladesh)*

“In 2003, the WHO recommended a low osmolality ORS (ORSaline–N) for childhood diarrhea management given its superior efficacy. Adopting the recommendation, SMC switched to ORSaline–N and with the support of USAID built a manufacturing plant for mass production and national distribution ORS. Now, all public and private sector companies produce low osmolality ORS. The easy-to-use ORS packets available at a cheaper price played a key role in increasing ORS use in Bangladesh.” *(Informant from Bangladesh)*

## DISCUSSION

Despite substantial progress in preventing child deaths since 1990, diarrhea was responsible for approximately 0.5 million such deaths globally in 2015 [1]. Among 129 surveys in low and middle-income countries since 2010 that assessed ORS use, the mean estimate of ORS use to treat children with diarrhea was 42% (with a range of 11%-94%). Since ORS is one of the most cost-effective interventions for preventing child deaths from diarrhea [40] and is a recommended intervention by Unicef and WHO [41] and under IMCI, this finding implies that there are ample missed opportunities to prevent many child deaths. This study suggests that a combination of strengthening the government, carrying out maternal and child health programs at the community level, and making these programs more affordable, can accelerate the use of ORS to treat childhood diarrhea on a population level. This is indicated by the associations identified in the study between increased ORS use, increased government effectiveness index, reduced out of pocket expenditures, and the increase in population coverage of the two maternal and child health interventions in the model: (1) four or more antenatal care visits and (2) care-seeking for pneumonia. There is some additional supportive evidence for these findings. From the time IMCI was introduced in the late to mid-1990s, at least until the mid-late 2000s when integrated community case management became a new focus, the program to control diarrheal diseases became more narrow, focusing on training first-level health facility workers, and losing much of the emphasis on diarrhea management in the home or availability of affordable ORS in the community (Fontaine O., personal communication) [42].

*The Success Factors study* found that health system improvements explained approximately half of global under-five mortality reduction from 1990-2010, while the improvements outside the health system–such as governance, water and sanitation, GDP per capita, women’s empowerment and education–explained the remainder [26,27]. Our study further supports that governance plays a vital role in improving child health, which is unsurprising considering governance is one of the six health system building blocks identified by WHO [43]. Political stability not making it into the final model only because government effectiveness and political stability are highly correlated. There is a strong association with ORS coverage and the political stability index in the univariable and multivariable analyses. Governance improvement is difficult to achieve, but efforts toward this end may result in improvements in child health when combined with other efforts. National governments and donors can interpret these findings to imply that accelerating the scale-up of key child health interventions, such as ORS, requires working at all levels of the health system from policies to community level programming. There also may be added synergies from increased financing (including decreased out-of-pocket expenditures) and improved government effectiveness [44].

Of the five health financing variables we tested, only out-of-pocket health expenditures remained significant in a multivariable model, and only weakly so. This finding implies a cautious interpretation that all of the health financing variables, such as total health spending in the country, affect ORS use by affecting access. Access to care was already adequately controlled for by the other variables in our model such as antenatal care and care-seeking for pneumonia. Financial limitations are, however, a potentially severe bottleneck in health care access, and our findings do not discount their importance.

The key informant interviews (KIIs) that we conducted further supports our interpretation. Representatives from Guinea-Bissau and Burundi confirmed the importance of effective government in improved ORS use. The respondents from Burundi stated that “ownership at all levels of the health system,” contributed to periods of expanded ORS use. In Guinea-Bissau, the respondents suggested that the reason ORS increased sharply from 2010-2014 was due to the deployment of community health workers in 2013 as part of a specific child health project in the country, and a decrease in political crises which would allow for more effective government index values. Informants from Bangladesh stressed the importance of national policies in increasing ORS use.

The significant spread of the random coefficient (**Figure 2**) and the contribution of time to improvements in ORS coverage (**Figure 3**) imply that, after controlling for the other factors in the model, approximately one-quarter of ORS improvements can be attributed to activities that were unmeasured and/or unique to the individual countries. The KIIs reflect such activities. A country seeking to reduce child diarrhea mortality dramatically–as Bangladesh achieved–could implement diarrhea-specific programming to accelerate progress.

### Limitations

Due to the limitations of this analysis, some caution is warranted. First, health indicators were only available for 235 data points. Cross-country regressions are known to be sensitive to non-random data availability, and while the statistical significance of several variables – such as government effectiveness and care-seeking pneumonia – were consistent; other variables were less consistently associated with improvements [43]. Second, these indicators are presumably proxies for health system strengthening; it is unlikely that increasing care-seeking for pneumonia will cause ORS coverage to also increase. Furthermore, national estimates do not always proxy well for subnational variation.

While the robustness of our findings, and the relatively high value for *R*^2^, increases our confidence–whether we included the full or restricted data set and after bootstrapping standard errors–we still may not have had sufficient statistical power to detect relationships for some of the other variables or they may be critical in omitted countries. Other variables that may be drivers of ORS coverage, such as community health worker density or severity of the diarrhea cases were not available and should be included in future analyses. We might expect ORS use to be higher where there are more trained health workers and in cases where the diarrhea was severe. Also, we were unable to include in our analysis information about the availability or stock of ORS, although key informants suggest that this would have been important to include in our model. Finally, while the longitudinal structure of our model strengthens our ability to make a causal inference, this remains a non-random retrospective analysis. Importantly, this analysis did not assess changes in zinc coverage, which has been shown to significantly impact morbidity when used in combination with ORS for treatment of diarrhea but often has significantly lower coverage [20]. Countdown to 2030 recommends integrating zinc with ORS in child health programming [44].

The variables dropped from the multivariable analysis are equally as important to note. GDP per capita, total fertility rates, clean water access, and maternal education all associated strongly with ORS coverage in univariable analyses (and maternal education is associated with ORS use in prior literature). However, these associations were not significant when controlling for other predictors of ORS coverage. While these indicators remain crucial for overall child mortality reduction, these findings imply that ORS coverage can be improved significantly even at low poverty or high fertility rates, probably through health system interventions (ie, deployment of community health workers, or demand generation) and improved governance.

Information provided by key informants was included to help the authors and readers interpret the finding of the analysis. The key informants were purposively selected from countries with relatively large changes in ORS coverage and therefore do not represent the full spectrum of countries in the study.

## CONCLUSION

Increasing ORS coverage can help prevent many child deaths between now and 2030, a target of Sustainable Development Goal 3. Much room for improving the treatment of childhood diarrhea with ORS remains, however. One path to increasing ORS coverage appears to lie mainly in reinvigorating known approaches to improving maternal and child health (demand generation, deployment of human resources and health promotion activities at the community level), and improved governance. Effective and affordable community health programs can play a vital role in improving the coverage of cost-effective child health services.

## Additional material

Online Supplementary Document
