# Supplementary material for: Strong community-based health systems and national governance predict improvement in coverage of oral rehydration solution (ORS): a multilevel longitudinal model
Source: J Glob Health. 2020 Feb 20;10(1):010503. doi: 10.7189/jogh.10.010503 (PMC7103061; doi:10.7189/jogh.10.010503)
Supplement: Online Supplementary Document [file jogh-10-010503-s001.zip › OSD Tables S1-2.docx]

**Supplementary Tables 1-2.**

**Supplementary Table 1. Hausman Test.**

Hausman test between fixed effects and random effects models. Random effects preferred.

---- Coefficients ----

| (b) (B) (b-B) sqrt(diag(V_b-V_B))

| fixed random Difference S.E.

-------------+----------------------------------------------------------------

time | .5955493 .6020193 -.0064699 .0123315

------------------------------------------------------------------------------

b = consistent under Ho and Ha; obtained from xtreg

B = inconsistent under Ha, efficient under Ho; obtained from xtreg

Test: Ho: difference in coefficients not systematic

chi2(1) = (b-B)'[(V_b-V_B)^(-1)](b-B)

= 0.28

Prob>chi2 = 0.5998

**Supplementary Table 2. Model selection for baseline models.**

Information criteria for testing different model specifications.

------------------------------------------------------------------------------------------------------

Model | Obs ll(null) ll(model) df AIC BIC

-------------+----------------------------------------------------------------------------------------

Full Model | 300 . -1112.041 11 2246.081 2286.823

No Rand. Coef.| 300 . -1116.994 9 2251.987 2285.321

-------------------------------------------------------------------------------------------------------

AIC = Akaike Information Criterion. BIC = Bayes Information Criterion. Rand coef = random coefficient.

Full model: unstructured covariances with autocorrelated residuals.

$$y_{ij}=\beta_{0}+\beta_{1}*t_{j}+{\beta X}_{ij}+\alpha_{k}+\mu_{i}+y_{i}*t_{j}+\varepsilon_{ij}$$
